# CEACAM6 is upregulated by *Helicobacter pylori* CagA and is a biomarker for early gastric cancer

**DOI:** 10.18632/oncotarget.10528

**Published:** 2016-07-11

**Authors:** Rony K. Roy, Michal M. Hoppe, Supriya Srivastava, Animesh Samanta, Neel Sharma, Kar Tong Tan, Henry Yang, Dominic C. Voon, Brendan Pang, Ming Teh, Naoko Murata-Kamiya, Masanori Hatakeyama, Young-Tae Chang, Wei Peng Yong, Yoshiaki Ito, Khek Yu Ho, Patrick Tan, Richie Soong, Phillip H. Koeffler, Khay Guan Yeoh, Anand D. Jeyasekharan

**Affiliations:** ^1^ Cancer Science Institute of Singapore, National University of Singapore, Singapore; ^2^ Department of Chemistry, National University of Singapore, Singapore; ^3^ Division of Gastroenterology and Hepatology, National University Hospital, Singapore; ^4^ Department of Pathology, National University Hospital, Singapore; ^5^ University of Tokyo, Tokyo, Japan; ^6^ Singapore Gastric Cancer Consortium, National University of Singapore, Singapore

**Keywords:** gastric cancer, biomarker, helicobacter pylori, CEACAM6, endoscopy

## Abstract

Early detection of gastric cancers saves lives, but remains a diagnostic challenge. In this study, we aimed to identify cell-surface biomarkers of early gastric cancer. We hypothesized that a subset of plasma membrane proteins induced by the *Helicobacter pylori* oncoprotein CagA will be retained in early gastric cancers through non-oncogene addiction. An inducible system for expression of CagA was used to identify differentially upregulated membrane protein transcripts *in vitro*. The top hits were then analyzed in gene expression datasets comparing transcriptome of gastric cancer with normal tissue, to focus on markers retained in cancer. Among the transcripts enriched upon CagA induction *in vitro*, a significant elevation of CEACAM6 was noted in gene expression datasets of gastric cancer. We used quantitative digital immunohistochemistry to measure CEACAM6 protein levels in tissue microarrays of gastric cancer. We demonstrate an increase in CEACAM6 in early gastric cancers, when compared to matched normal tissue, with an AUC of 0.83 for diagnostic validity. Finally, we show that a fluorescently conjugated CEACAM6 antibody binds avidly to freshly resected gastric cancer xenograft samples and can be detected by endoscopy in real time. Together, these results suggest that CEACAM6 upregulation is a cell surface response to *H. pylori* CagA, and is retained in early gastric cancers. They highlight a novel link between CEACAM6 expression and CagA in gastric cancer, and suggest CEACAM6 to be a promising biomarker to aid with the fluorescent endoscopic diagnosis of early neoplastic lesions in the stomach.

## INTRODUCTION

### The clinical need for an endoscopic marker of early gastric cancer

Gastric cancer is a leading cause of mortality from neoplastic disease, and is particularly prevalent in Asian populations [1]. Symptomatology is rare and non-specific in the early stages of the disease [2] necessitating screening by endoscopy to differentiate benign gastric conditions from cancer. Early diagnosis of gastric cancer through endoscopic methods, and its treatment by submucosal resection has enabled 5-year survival rates of up to 95% in cohort studies [3, 4]. In contrast, the overall 5-year survival of all patients with gastric cancer, as determined from the NCI Surveillance Epidemiology and End Results database *(http://seer.cancer.gov)* is only 26%, as many of the patients have regional or distant spread at the time of diagnosis. This has resulted in the categorization of ‘Early Gastric Cancer’ (EGC) as a unique entity, defined by T1 disease, and good clinical outcomes. However, early diagnosis of EGC and the premalignant stage, gastric intestinal metaplasia (GIM), is not trivial using conventional endoscopic techniques. There is therefore an urgent need for improved methods to facilitate early diagnosis of cancer and pre-malignant lesions of the stomach, and thereby ensure early and effective treatment.

The primary method of diagnosis of gastric cancer and pre-malignant conditions relies on accurate endoscopic visualization and tissue sampling through biopsies. The current gold standard is white light endoscopy, however this is fraught with limitations, primarily due to reliance on the skill of the operator in terms of adequate visualization and tissue sampling. Early cancers and dysplastic lesions are often not obvious to visual inspection, and require years of experience to identify [5]. Furthermore, variation in disease prevalence has led to variations in endoscopic skill levels in different parts of the world, with missed diagnosis rates of 8-35% [6, 7]. The development of methods to improve the contrast between normal and early cancerous tissue is likely to have a significant benefit in the global management of this disease. Several image-enhanced tools currently aid in making the diagnosis of gastric cancer [8, 9], including narrow band imaging and auto fluorescence imaging, which rely on the inherent fluorescent properties of cancer tissue. However, these techniques are not adequately sensitive and specific for the routine diagnosis of early gastric cancers. Methods that rely on or exploit the molecular differences in normal and abnormal tissue are not widely studied in the diagnosis of early gastric cancer [10]. By utilizing cell surface markers combined with a fluorescent probe, we hypothesize greater image capturing capabilities for the real-time diagnosis of early gastric cancer and ultimately real-time treatment. This is particularly attractive in view of the move towards screening of pre-neoplastic conditions, where targeted biopsy sampling as opposed to random biopsies is likely to achieve optimum cost-effectiveness for patients.

### Design of a screen for cell surface markers of early gastric cancer

Proteins reported to be overexpressed on the surface of advanced gastric cancers include SLC3A2 [11], CDH17 [12], EPHA2, FGFR2 [13], and CD44v8-10 [14]. To focus our search on putative membrane protein markers that are elevated in early gastric cancers, we designed a screen based on known molecular events in early stages of gastric carcinogenesis. Gastric adenocarcinomas are strongly associated with *Helicobacter pylori* infection [15] and chronic gastritis [1]. The *H. pylori* CAG pathogenicity island codes for a type IV secretion system, which injects the CagA protein from the bacterium into the host epithelium [15]. Once in the host cell, CagA dysregulates a number of key pathways controlling proliferation, differentiation and polarity [16], initiating the process of carcinogenesis. We hypothesized that at least a fraction of the membrane proteins upregulated by the presence of CagA are likely to be retained in gastric cancer through the principle of non-oncogene addiction [17]. To focus on CagA related changes that are markers of cancer (not of infection or hyperplasia), a second ‘virtual’ screen was performed in existing mRNA expression databases of gastric cancer in comparison to normal tissue.

Here the results of this 2-step screening approach are reported, highlighting the identification of CEACAM6 as a potential endoscopic biomarker of early gastric cancer. We show that the cell surface protein CEACAM6 is upregulated by the *H. pylori* CagA oncoprotein, and highly expressed on early gastric cancers as well as premalignant lesions. A fluorescently tagged antibody to CEACAM6 avidly binds to gastric cancer tissue and can be visualized by commercially available endoscopic methods.

## RESULTS

### Two-step screening identifies CEACAM6 to be upregulated by CagA, and retained in gastric cancer

An ideal screening methodology using cell lines is one where the experimental and control groups differ by a single acute perturbation, to minimize chronic adaptation in culture. MKN28 gastric epithelial cells [18] containing a stable CagA expression cassette under a tetracycline response element [19] were used in our screen (WT-A10 cells). These cells retain the capacity to dysregulate β-catenin [19], and induce the expression of key intestinal transdifferentiation markers (CDX1 and MUC2) on CagA expression, confirming the line as a valid model for the preliminary phase of the screen. The expression of CagA upon induction in this cell line was confirmed using RT-PCR, and these cells reproduced the phenotypic changes previously described for *in vitro* CagA expression (Supplementary Figure 1).

For the screen, expression of CagA was induced by the withdrawal of doxycycline for 48h, followed by gene expression array analysis to identify differentially regulated genes. Our analysis focused on those transcripts that putatively code for intrinsic, extrinsic, integral and anchored membrane proteins, based on gene ontology. The top ten differentially upregulated membrane protein transcripts identified in this cellular assay (Table 1) were interrogated in a gene expression cohort of gastric cancers and normal controls [20]. Among the hits identified in the CagA screen, 4 of them (MMP1, CEACAM6, ITGA2, C3) showed at least 2-fold increases in gastric cancer when compared to normal controls (Table 1).

**Table 1:** CagA regulated cell surface proteins and their expression in gastric cancer

UniGene	Gene	Description	WT-A10 (CagA +/−) Microarray		SGCC GC Microarray	
			Fold Change	p-value	Fold Change	p-value
**Hs.201877**	TMPRSS11E	Transmembrane protease, serine 11E	10.27	1.95E-04	0.97	1.96E-01
**Hs.368912**	DPP4	Dipeptidyl-peptidase 4	2.97	9.77E-04	1.70	3.12E-05
**Hs.83169**	MMP1	Matrix metallopeptidase 1 (interstitial collagenase)	2.88	4.11E-03	2.18	1.46E-06
**Hs.525105**	SLITRK6	SLIT and NTRK-like family, member 6	2.70	1.21E-02	1.16	4.02E-02
**Hs.86447**	TNFRSF9	Tumor necrosis factor receptor superfamily, member 9	2.67	5.64E-04	0.95	2.18E-02
**Hs.466814**	CEACAM6	Carcinoembryonic antigen-related cell adhesion molecule 6	2.53	5.76E-04	8.06	9.36E-19
**Hs.631594**	LYPD3	LY6/PLAUR Domain Containing 3	2.49	8.73E-04	1.15	6.08E-03
**Hs.247879**	C6orf25	Chromosome 6 open reading frame 25	2.30	6.56E-03	0.91	6.35E-08
**Hs.369520**	SYTL2	Synaptotagmin-like 2	2.30	2.92E-02	0.70	1.44E-06
**Hs.482077**	ITGA2	Integrin, alpha 2 (CD49B, alpha 2 subunit of VLA-2 receptor)	2.23	1.03E-04	2.32	8.53E-24
**Hs.647419**	CD68	CD68 molecule	2.19	1.97E-03	1.16	7.48E-05
**Hs.529053**	C3	Complement Component 3	2.19	1.19E-02	2.08	1.01E-10

A list of putative cell surface proteins transcriptionally upregulated by overexpression of CagA-microarray analysis performed on the Gene 1.0 ST platform using an inducible *cagA* cell line. Top hits were screened through microarray databases of Gastric Cancer vs. normal tissue from the Singapore Gastric Cancer Consortium (SGCC) GSE15460 (Normal=85, Tumor=189).

**Figure 1:**
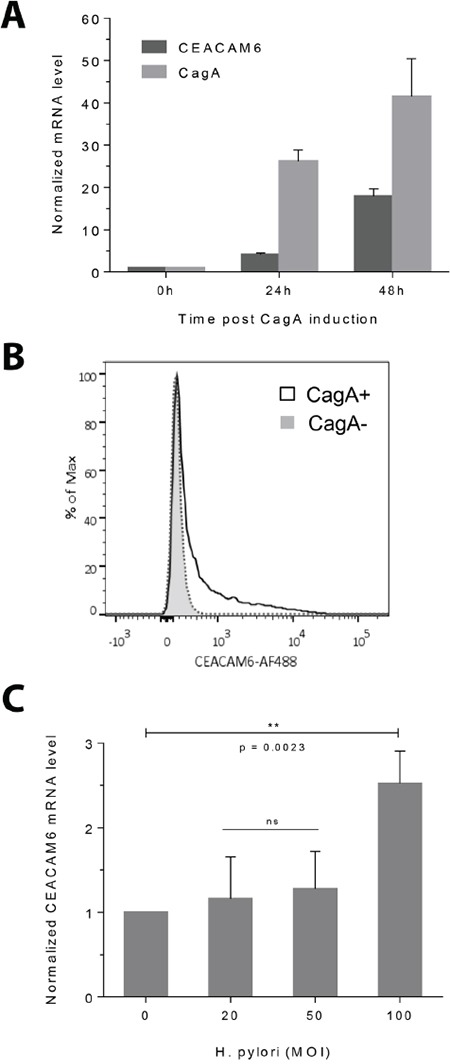
CEACAM6 expression is upregulated by CagA and *H. pylori* infection **A.** RT-PCR showing the time course of CEACAM6 transcript upregulation after CagA induction, in relation to CagA levels in the CagA inducible WT-A10 cell line. CT values are normalized to 18S RNA and shown relative to CEACAM6 or CagA levels before CagA induction to demonstrate fold change. Mean±SD, n=3. **B.** Flow cytometric histogram of CEACAM6 protein levels in CagA-negative (baseline) and CagA-positive (induced) cells, showing elevated CEACAM6 protein levels 48h after CagA induction. **C.** RT-PCR showing that *H. pylori* infection upregulates CEACAM6. MKN28 cells were infected with increasing MOI's of isogenic wild-type *H. pylori* and CEACAM6 transcripts were measured by RT-PCR 24h after infection. Mean±SD, n=3. Unpaired t test, ns = not significant.

**Figure 2:**
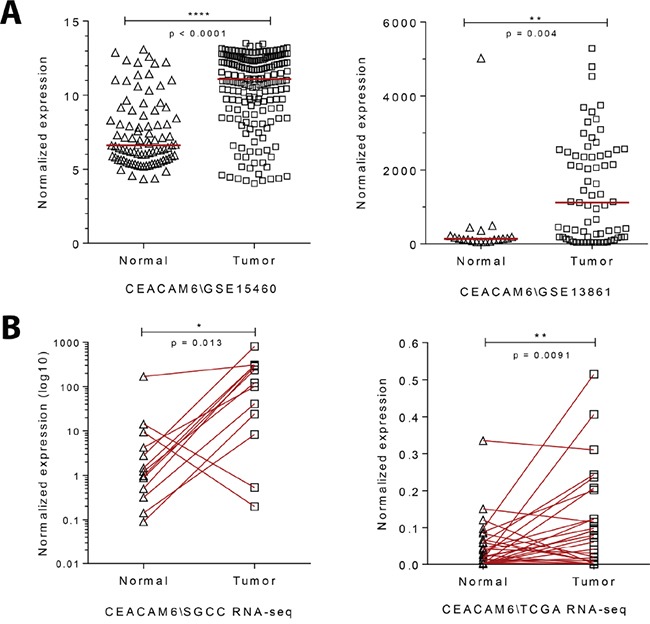
Gene expression analysis of gastric cancers show an enrichment for CEACAM6 **A.** Dot plot of relative CEACAM6 transcript levels in gene expression arrays of gastric cancer in comparison to normal stomach tissue (GSE15460 (SGCC) – Normal=85, Tumor=185; GSE13861 - Normal=19, Tumor=65). Median, unpaired t test. **B.** Plot of relative CEACAM6 transcript levels using RNA-seq analysis of matched gastric cancers compared to normal adjacent tissue (SGCC n=15, TCGA n=29). Paired t test.

CEACAM6 was chosen for further analysis on the basis of its increased levels in gastric cancer over normal tissue (8-fold in the SGCC data set). RT-PCR analysis on the same MKN28 CagA inducible cell line showed a time-dependent increase in CEACAM6 transcripts with CagA induction, peaking at 48h post induction in this system (Figure 1A). Levels of the CEACAM6 protein were also higher after CagA induction, as evidenced by flow cytometry analysis using an antibody to CEACAM6 (Figure 1B). Furthermore, infection of wild-type MKN28 cells by a live CagA wild-type *H. pylori* strain also resulted in an increase in CEACAM6 transcripts (Figure 1C), confirming this association to be physiological and not an artifact of high levels of overexpression in the tet-inducible system. The high expression of CEACAM6 transcripts in gastric cancer was confirmed using other publicly available datasets of gastric cancer gene expression [21–24] (Figure 2A). Evaluation of CEACAM6 transcript levels using RNA-seq data of matched tumor and adjacent normal tissue, from the SGCC (n=15) and TCGA (n=29) also confirmed a significant enrichment of the transcript in gastric cancer (Figure 2B). Together, these data suggest that CEACAM6 mRNA is upregulated by the presence of *H. pylori* CagA, and is retained in advanced gastric cancers.

### Quantitative histopathology confirms CEACAM6 upregulation in gastric cancers compared to regions of matched adjacent normal tissue

The Vectra 2 multispectral microscopy platform allows a quantitative assessment of antibody staining in histological samples [25]. First, this multispectral imaging system allows creation of a unique spectral profile for every chromogen used with the sample [26], thereby enabling the ‘unmixing’ of the chromogen signal from a counterstain (Supplementary Figure 2). The unmixed chromogen signal can be quantitated in terms of mean optical density per unit area of the tumor/normal tissue region, to give a numerical readout of protein expression, which we demonstrate to correlate well with pathologist scoring for a control marker (Supplementary Figure 3).

**Figure 3:**
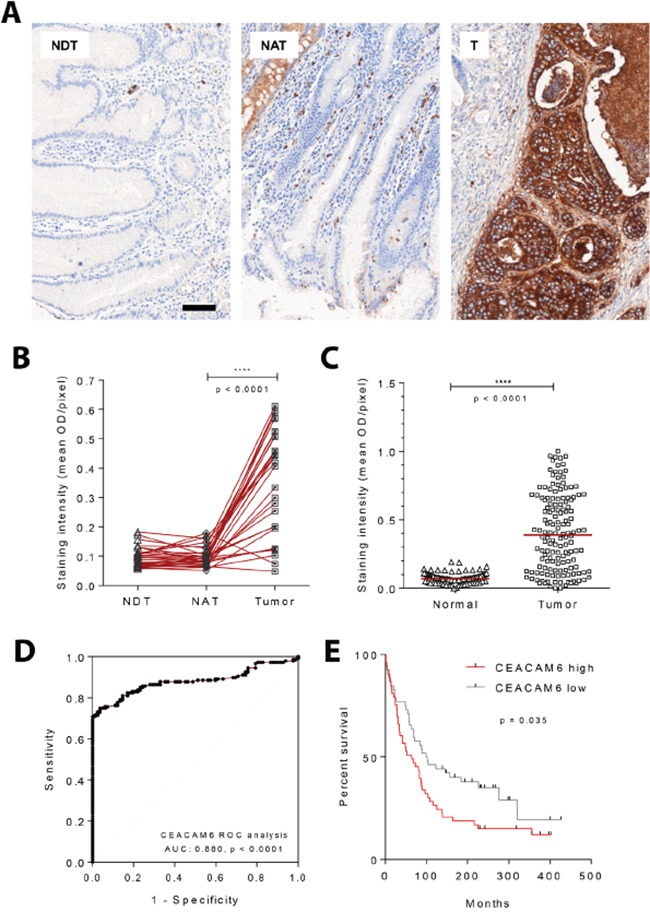
CEACAM6 protein expression by quantitative immunohistochemistry in gastric cancers **A.** Representative images acquired from a tissue microarray of gastric cancers and matched normal tissues stained for CEACAM6 using standard immunohistochemical methods. Scale bar=50μm. **B.** Images obtained as depicted in A were quantified using the Vectra 2 system. Commercially available gastric adenocarcinoma tissue microarray consisting of 29 cases of matched normal distant tissue (NDT), normal adjacent tissue (NAT) and tumor (T), shows stronger CEACAM6 staining within regions of cancer compared to adjacent normal tissue. Paired t test. **C.** Upregulation of CEACAM6 protein in gastric cancer was also confirmed using a local gastric adenocarcinoma tissue microarray (National University Hospital, Singapore). Normal=82, Tumor=158. Median, unpaired t test. **D.** ROC analysis was performed on tissue array set shown in C, which yielded an AUC of 0.88 for the diagnosis of gastric cancer using CEACAM6 IHC. **E.** Overall survival analysis of 106 gastric cancer patients. Cases were divided into CEACAM6 high/low groups across the median value, based on scoring readout shown in C. CEACAM6 high group shows a significant trend towards poorer overall survival. Log-rank (Mantel-Cox) test.

Accordingly, this platform was used to confirm the overexpression of CEACAM6 protein in a tissue microarray of advanced gastric cancer with matched normal tissue (n=29). Consistent with earlier reports [27], a significant increase in CEACAM6 immunostaining for gastric cancer in comparison to adjacent normal tissue was noted (Figure 3A and 3B), p < 0.001. A larger in-house tissue microarray generated at National University Hospital, Singapore, also showed a significant enrichment of CEACAM6 expression in tumor cores (Figure 3C), p < 0.001. A receiver-operative characteristic (ROC) analysis of the larger tissue microarray yielded an area under the curve (AUC) of 0.880 for the discrimination of cancer from normal samples by CEACAM6 (Figure 3D). This group of samples was further divided into CEACAM6 “high/low” groups across the median value, based on the staining intensity readouts depicted in Figure 3C, to perform overall survival analysis. The CEACAM6 “high” group of patients showed a trend towards poorer overall survival (Figure 3E). Median staining intensity numerically increased with tumor stage, however it is not significantly different between respective tumor stage groups. Furthermore, CEACAM6 high staining cases were not associated with any specific tumor morphology or tumor grade (Supplementary Figure 4).

### CEACAM6 levels are elevated in early gastric cancer and dysplastic lesions

**Figure 4:**
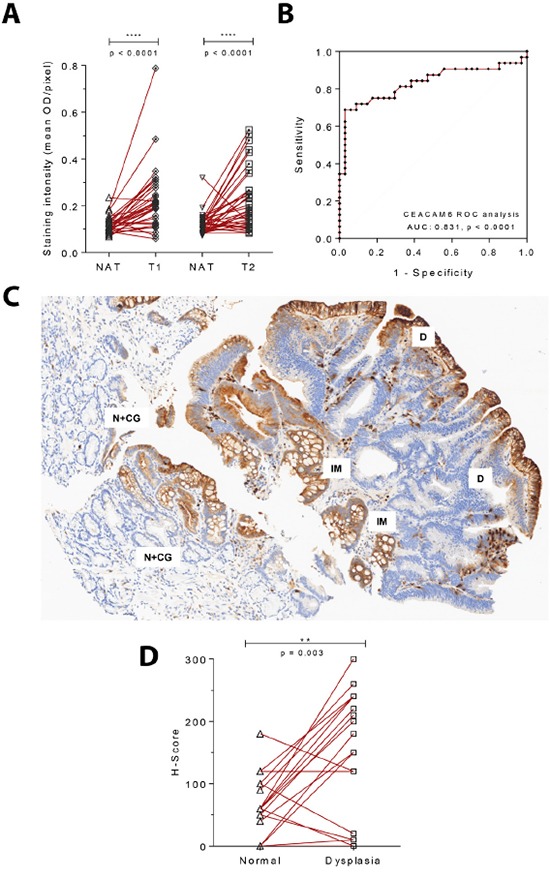
CEACAM6 is upregulated in both early gastric cancers and dysplastic lesions of the stomach **A.** CEACAM6 IHC staining of an early gastric adenocarcinoma tissue microarray with matched normal adjacent tissue (clinical stage 1 - T1N0M0 and T1N1M0=32, T2N0M0=39). Paired t test. **B.** ROC analysis of CEACAM6 staining in T1 early gastric cancer shows an AUC of 0.831 for the identification of early gastric cancer from adjacent normal tissue. **C.** A representative image of CEACAM6 staining in a region of dysplasia depicting increase in staining intensity from normal gastric glands showing mild chronic gastritis (N+CG) to intestinal metaplasia (IM) to dysplasia (D). Scale bar=100μm. **D.** Quantitation of the H-Score for CEACAM6 in 20 cases of dysplasia with adjacent normal tissue performed by a histopathologist. A significant increase in CEACAM6 staining is observed in dysplastic regions. Paired t test.

As CEACAM6 levels increase in response to *H. pylori* CagA (an early event in carcinogenesis) and are retained in advanced cancers, we hypothesized that it may be a marker of early gastric cancer. To test this hypothesis we obtained a commercial tissue microarray of early gastric cancers (clinical stage I, n=70) with matched adjacent normal tissue. This microarray was stained for CEACAM6, and quantitated on the Vectra 2 platform as described earlier. We observed a clear increase in the mean staining intensity for CEACAM6 in early gastric cancers (T1N0M0 and T1N1M0) when compared to adjacent normal tissue (Figure 4A), p < 0.001. CEACAM6 staining provided an AUC of 0.831 in an ROC analysis for the distinction of normal mucosa from early gastric cancer (Figure 4B).

In a cohort of dysplastic gastric lesions, a significant increase in CEACAM6 expression levels was noted, in comparison to matched normal tissue within the section (Figure 4C). Of 20 cases of dysplasia (10 high grade and 10 low grade), 7 and 8 cases respectively showed a complete spectrum of states from normal to intestinal metaplasia (IM) to dysplastic mucosa. An overexpression of CEACAM6 in the dysplastic area was observed, with a significant increase in the H-Score for CEACAM6 staining compared to adjacent normal gastric mucosa, p = 0.003 (Figure 4D). The quantification of CEACAM6 scores for dysplastic lesions was done by an experienced histopathologist, to avoid errors from the automated Vectra 2 algorithm due to subtle features of these lesions. Interestingly, the expression of CEACAM6 was highest in the IM area as compared to the adjacent normal and dysplastic mucosa. In contrast, minimal staining of CEACAM6 occurred in regions of chronic gastritis, the most prevalent lesion associated with *H. pylori* infection [28] (Figure 4C).

Together, these findings suggest that CEACAM6 may be useful as a surface marker of early gastric cancer and pre-neoplastic lesions. The marked increase in immunoreactivity to CEACAM6 in dysplastic and early neoplastic lesions over adjacent normal tissue is promising for the development of a biomarker for endoscopy, where a significant challenge is identifying the correct region to biopsy.

### A fluorescently labeled antibody to CEACAM6 binds avidly to gastric cancer tissue *ex vivo*

**Figure 5:**
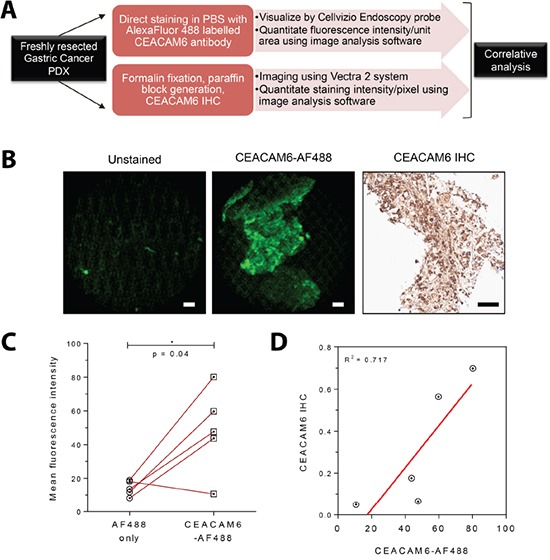
*Ex vivo* staining of a fluorescently conjugated antibody to CEACAM6 and visualization with a confocal laser endomicroscopy probe **A.** Schematic diagram of the experimental plan. Freshly resected patient derived xenografts (PDXs) of gastric cancer were divided into two pieces - one section was used for direct fluorescent staining and the remainder for immunohistochemistry. **B.** Representative images from freshly resected gastric cancer xenograft tumors without fixation showing clearly visible fluorescence signal from the CEACAM6 antibody conjugated to Alexa Fluor 488 (imaged with a Cellvizio confocal laser endomicroscopy probe, compatible with commercial endoscopy equipment). CEACAM6 IHC staining of the same xenograft is shown for reference. Scale bar=50μm. **C.** Analysis of signal intensity of CEACAM6-AF 488 stained tumors in comparison to unstained controls, as shown in B, n=5. Paired t test. **D.** Correlative analysis of Cellvizio CEACAM6-AF 488 fluorescence intensity values with CEACAM6 IHC staining intensity, as shown in B, n=5.

A surface protein such as CEACAM6 could serve as a marker to identify gastric malignant pathology, target biopsies to the right site, and provide a means of lesion resection in real time. As a proof of concept for the potential utility of CEACAM6, we aimed to evaluate the ability of a conjugated anti-CEACAM6 reagent to directly bind gastric cancer tissue. A monoclonal antibody to CEACAM6 was conjugated covalently to Alexa Fluor 488 and purified through size exclusion chromatography (Supplementary Figure 5A). The ability of this reagent to bind to five freshly resected patient derived xenografts (PDX) of gastric cancer in a direct staining assay was tested (experiment schematic in Figure 5A). To simulate the setting of utilizing this marker during endoscopy, fluorescent images of the stained PDX samples were obtained using a clinically approved Cellvizio confocal endomicroscopy probe (Mauna Kea Technologies), which is compatible with commercial endoscopy systems. We demonstrate low background signal from unstained tumor tissue with strong and clear fluorescent signal from tissue samples that have been exposed to anti-CEACAM6 Alexa Fluor 488 conjugated antibody for 30 minutes at room temperature (Figures 5B and 5C, Supplementary Figure 5B). A video recording of the CEACAM6 signal obtained from the resected gastric PDX tissue using the confocal probe offers a representation of the endoscopic visualization of tumor tissue (Supplementary movie 1). Since PDX models do not have an appropriate negative control of normal tissue with human CEACAM6, we correlated the fluorescent signal of the Alexa Fluor 488 anti-CEACAM6 conjugate with the amount of CEACAM6 estimated for each tumor by quantitative histopathology. Each tumor was divided into 2 sections, one of which was stained directly with the fluorescent antibody, and the other was fixed for staining with routine IHC analysis. A correlation between the two data sets was observed, moreover a tumor negatively staining for CEACAM6 by IHC also showed minimal staining with the fluorescently conjugated reagent (Figure 5D, Supplementary Figure 5B). Together, these results suggest that a fluorescently labelled CEACAM6 antibody can directly bind to live gastric cancer tissue and therefore have applicability in the context of endoscopic diagnosis of early gastric cancer.

## DISCUSSION

In this paper, we aimed to identify a candidate cell-surface biomarker for use in fluorescence endoscopy for the diagnosis of early gastric cancer. We chose to use a CagA based screen, since CagA exogenous expression in the epithelium is sufficient for development of gastric cancer in mice, even in the absence of *H. pylori* infection [29], confirming its activity as a genuine oncoprotein. Indeed, despite the prevalence of *H. pylori* infection in the general population, a significant increase in CAG pathogenicity islands in cancer is noted when compared to dysplastic or normal tissue [30]. Generation of a cell line that inducibly expresses the CagA protein [19] allowed a controlled, cell-based assay to identify CEACAM6 as a novel cell-surface target upregulated by the oncogene.

CEACAM6 has previously been reported to be upregulated in a variety of cancers, including gastric cancer [27] but its biological role remains unclear. It is a cell-adhesion protein, capable of a variety of homotypic and heterotypic interactions, mediating cell-cell interactions. The binding partners of CEACAM6 are not well characterized, but it has been described to promote *in vivo* metastasis, tumor angiogenesis, vasculogenic mimicry in gastric cancer, and confers a poor prognosis to cancers overexpressing the protein [31]. Here, we demonstrate that it is highly expressed in pre-malignant lesions of the stomach and early gastric cancer, with significant clinical implications for early diagnosis and targeting of biopsies. The lower expression of CEACAM6 in regions of chronic gastritis is also significant, as a putative marker would ideally need to distinguish pre-neoplastic lesions from gastritis, which is far more prevalent in patients with *H. pylori* [28]. The ‘hit and run’ model [32] for *H. pylori* carcinogenesis, whereby *H. pylori* infection may be a transient initiating event, suggests that while CagA is critical to the onset of gastric carcinogensis, it is not always required for maintenance of the oncogenic phenotype. The pro-oncogenic actions of CagA are supplanted by a series of other pathway alterations that continue the process of carcinogenesis [32], and our data suggest that CEACAM6 may be one of these altered pathways that is retained through non-oncogene addiction [17].

The few reports that exist on marker-guided gastroscopy focus on the use of fluorescently conjugated antibodies to carcinoembryonic antigen (CEA) or MUC1 [33], with limited clinical validation. A marker that highlights early lesions could decrease the chances of false negative biopsies, and enabling reliable follow up. We show that a fluorescently conjugated antibody to CEACAM6 is able to bind to gastric cancer samples from xenografts, and be visualized by a confocal laser endomicroscopy (CLE) probe that is compatible with most commercially available endoscopy equipment. While CLE imaging on its own has been shown promise in identifying superficial gastric neoplasia [35], it requires significant time to cover the entire surface area of the stomach for a thorough evaluation. In our study, we demonstrate that a fluorescent marker to the cell surface protein CEACAM6 could complement this technique, by providing a ‘beacon’ to high risk regions that may be assessed further either by confocal imaging or histological analysis after biopsy. The compatibility of the Cellvizio system with existing equipment will facilitate clinical research to confirm its utility in this setting. There are several fluorophores approved by the FDA for human use [34] but concerns remain about their efficacy in the acidic pH of the native stomach, and the ability of an antibody conjugate to penetrate through the mucin layer. However, cancers often disrupt the mucin layer, and the pH can be altered through pre-treatment with proton pump inhibition, allowing for the design of trials to evaluate such reagents in early gastric cancer diagnosis. The high levels of discrimination demonstrated by the AUC of CEACAM6 in early gastric cancer and intestinal metaplasia specimen suggest a rationale for the further development of this reagent as a tool for early gastric cancer diagnosis, in conjunction with advances in fluorescent endoscopy.

In summary, this work uses a CagA based *in vitro* screen which identified CEACAM6 as a novel biomarker of early gastric cancer and dysplastic lesions, and demonstrated the ability of a commercial endoscopy system to detect fluorescently labelled CEACAM6 reagent in *ex vivo* preparations of gastric cancer. The results show a potential clinical utility of a fluorescently conjugated CEACAM6 antibody for the endoscopic diagnosis of early gastric cancer.

## MATERIALS AND METHODS

### Cell culture, *H.pylori* infection, qPCR and flow cytometry

WT-A10 cell line is a MKN28-derived stable transfectant clone that inducibly expresses CagA using a tet-off system [19]. WT-A10 cells were cultured in RPMI 1640 medium supplemented with 10% fetal bovine serum (FBS), 10 mM HEPES, 1 mM sodium pyruvate, 0.1 mM non-essential amino acid, penicillin G sodium & streptomycin sulfate, 2 mM L-glutamine, 0.5 mg/ml G418, 0.1 mg/ml hygromycin B and 1 μg/ml Doxycycline. MKN28 human gastric epithelial cells were cultured in RPMI 1640 medium supplemented with 10% FBS.

The *H. pylori* isogenic wild-type strain (NCTC11637) was cultured in Trypticase soy agar with 5% sheep blood (BD Biosciences) at 37°C in humidified and microaerophilic atmosphere to form colonies. The colonies were inoculated in Brucella broth (Sigma-Aldrich) with 10% FBS for 24h to prepare broth cultures. Gastric cancer cell lines were infected with broth-cultured *H. pylori* at 0, 20, 50 and 100 MOI for 24h.

Total RNA was extracted using the RNeasy Mini Kit (Qiagen), and cDNA was synthesized with the iScript RT kit (Bio-Rad Laboratories) according to the instructions from the manufacturer. qPCR was performed with the KAPA SYBR FAST qPCR Kits (Kapa Biosystems, Wilmington, MA) using CEACAM6 specific oligonucleotide primers (GACCCTCACTCTACTCAGC and CAGATTTTCCCCTGGACG) for SYBR Green-based measurements. Data analyses were performed on ABI Prism 7500 real-time PCR system (Applied Biosystems) and gene expression data were normalized to 18S or GAPDH levels.

CagA induced and non-induced WT-A10 cells were incubated with anti-CEACAM6 antibody (9A6, Abcam) or AF488 tagged anti-mouse IgG and counterstained with 1 ug/ml of propidium iodide after fixation. Cells were analyzed on a BD LSRII flow cytometer (BD Biosciences). Flow cytometry data were analyzed using the FlowJo computer software package (Tree Star, USA).

### Microarray analysis

RNA from WT-A10 cells was hybridized on Affymetrix GeneChip Human Gene 1.0 ST array. Following hybridization, array was washed and stained according to the standard Affymetrix protocol using Fluidics Station 450, GeneChip Scanner 3000 7G was used to measure the fluorescence intensity emitted by the labelled target. The raw data was subjected to further processing using the Affymetrix GeneChip Operating Software. Top hits obtained from CagA microarray analysis were screened through gastric cancer dataset from the Singapore Gastric Cancer Consortium (SGCC), GSE15460, and GSE13861 (PMID: 21447720). RNA-seq datasets were obtained from SGCC (matched, n=15) and TCGA (matched, n=29).

### Immunohistochemistry and quantitative analysis

Tissue microarray of gastric cancers (Normal=82, Tumor=158, matched n=46), as well as low and high grade dysplasia (n=20) were obtained from National University Hospital, Singapore. Tissue microarrays obtained from US Biomax Inc., Rockville, USA consisted of gastric adenocarcinoma (HStm-Ade090PG-01; normal distant tissue, normal adjacent tissue and tumor, n=29, 3 cores/case) and early gastric adenocarcinoma (HStm-Ade150CS1-01; T1N0M0=29, T1N1M0=3, T2N0M0=39 and matched normal adjacent tissue, 2 cores/case).

Slides were deparaffinized in xylene three times for 5 minutes and rehydrated in an ethanol gradient 100%, 90%, 70% and H_2_O for 5 minutes each. Pressure antigen retrieval was performed in citrate antigen retrieval buffer pH 6.0 (Dako) using a Retriever device (Electron Microscopy Sciences). Slides were stained using a standard automated immunohistochemistry protocol (Bond-Max, Leica Biosystems). Briefly, slides were blocked for 30 minutes with antibody diluent (Bond Primary Antibody Diluent, Leica Biosystems) and subsequently incubated with CEACAM6 antibody for 30 minutes. Detection was performed using a polymer HRP detection system (Bond Polymer Refine Detection, Leica Biosystems) and 3,3′-diaminobenzidine (DAB) was used for visualization. Slides were incubated with hematoxylin to serve as a counterstain.

Acquisition and image analysis was done with the Vectra 2 multispectral automated imaging system (PerkinElmer) and inForm 2.0 image analysis software, an interactive image segmentation system, as described in previous studies [26]. Nuance software (PerkinElmer) was employed to build the spectral libraries for the chromogens (DAB and hematoxylin). These chromogen signature profiles were later used to spectrally unmix and quantitate CEACAM6 staining intensity, with appropriate regions for analysis chosen by a pathologist.

### Antibody conjugation and confocal laser endomicroscopy

Briefly, 25μg of antibody was purified from carrier and mixed with bicarbonate buffer. The purified antibody was conjugated to Alexa Fluor 488 (final concentration 120μM) at room temperature and incubated in the dark. The conjugated antibody was purified using Bio-Gel P-30 size exclusion chromatography (Bio-Rad, chromatography, 90-180μm wet bead size).

Gastric cancer patient derived xenografts (PDX, n=5) were obtained from the Xenograft Cancer Models Facility (Cancer Science Institute of Singapore, NUS) and transferred to sterile phosphate buffered saline (PBS) immediately after resection. Each sample was divided into two: one part was used for formalin-fixation and IHC analysis of CEACAM6 levels as described above, and the second part was stained using Alexa Fluor 488 conjugated CEACAM6 antibody (1:100 dilution) for 30 minutes. Normal murine stomach tissue served as a negative control. After routine washes, dynamic images were recorded using a hand-held confocal endomicroscopy (Mauna Kea Technologies) probe directly applied both to the internal part and several areas of surrounding tissue.

## SUPPLEMENTARY INFORMATION




