# Supplementary material for: CEACAM6 is upregulated by Helicobacter pylori CagA and is a biomarker for early gastric cancer
Source: Oncotarget. 2016 Jul 11;7(34):55290–301. doi: 10.18632/oncotarget.10528 (PMC5342417; doi:10.18632/oncotarget.10528)
Supplement: Supplementary file 1 [file oncotarget-07-55290-s001.pdf]

CEACAM6 is upregulated by *Helicobacter pylori* CagA and is a biomarker for early gastric cancer

SUPPLEMENTARY INFORMATION

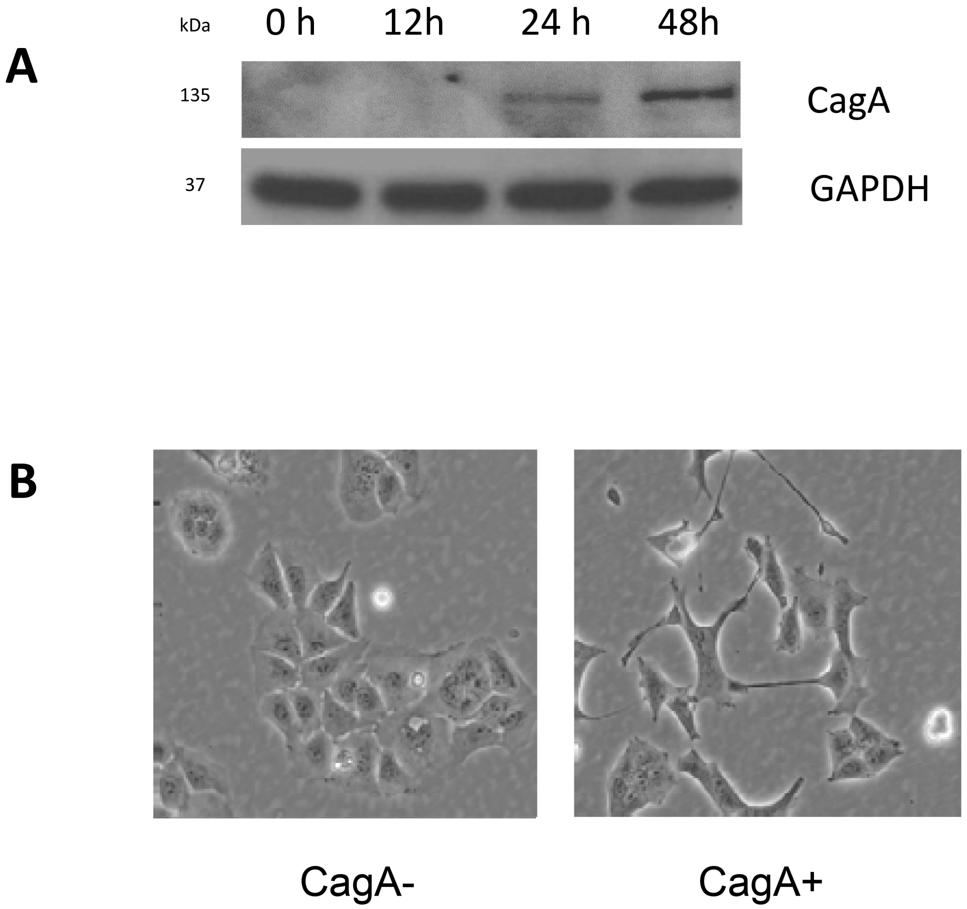

Supplementary Figure S1: A. Western blot demonstrating an increase in CagA protein expression using the doxycycline inducible tet-off system in WT-A10 cells. Haemagglutinin tagged CagA was detected using an antibody to HA. GAPDH serves as the loading control. **B.** Phase contrast microscopic image demonstrating characteristic 'hummingbird like' phenotypic changes observed in cell culture upon induction of CagA expression.

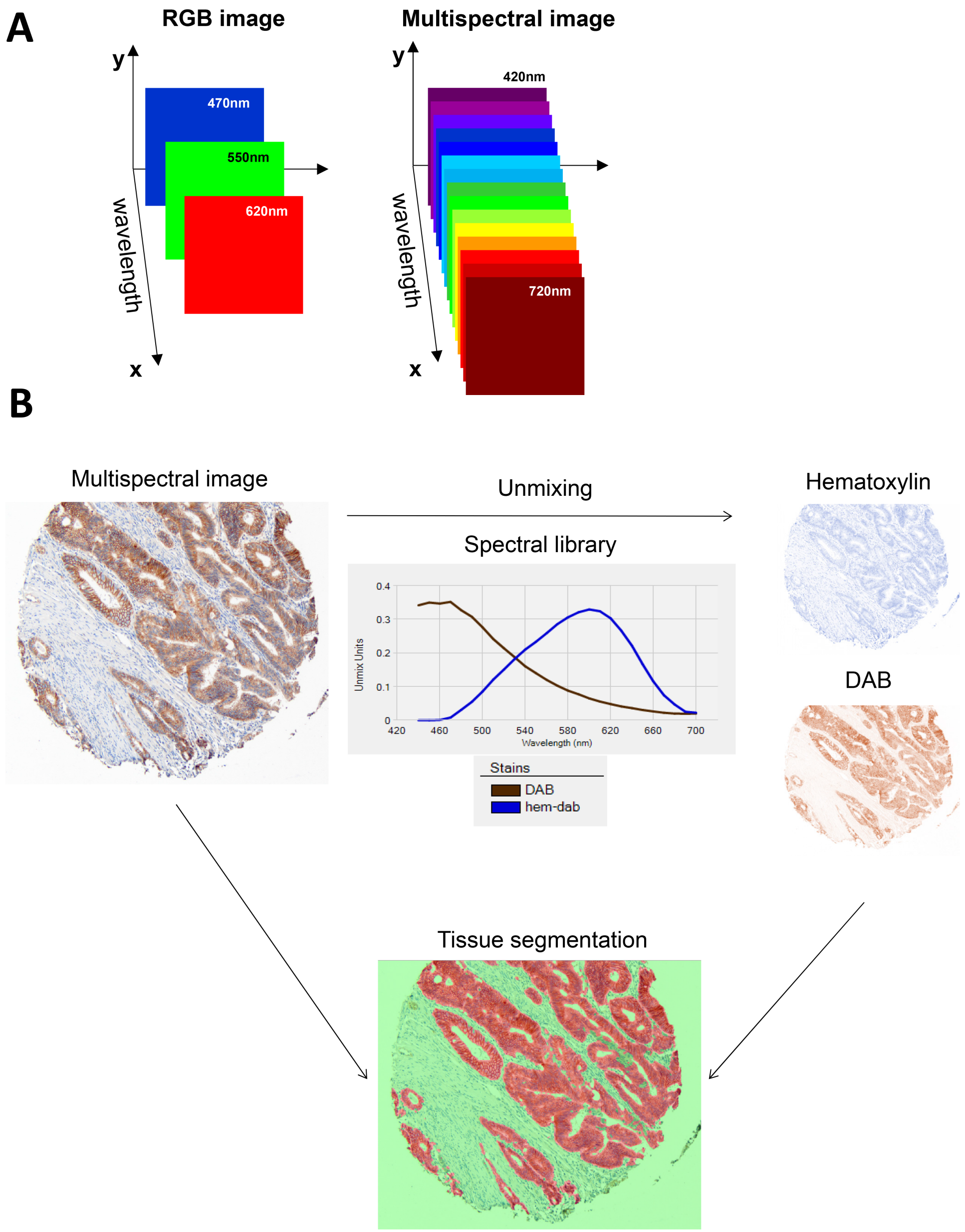

Supplementary Figure S2: A. Representation of differences between traditional microscopy and spectral imaging. The graphic on the left shows the typical 3 colour RGB (red-green-blue) image obtained by a standard colour camera. The graphic on the right represents the nature of images obtained by a spectral camera across the entire visible spectrum. **B.** Workflow of unmixing and analysing a spectrally acquired image using the Vectra 2 platform. A multispectral image can be unmixed into its constituent colours, as each colour has a unique spectral signature across the visible wavelength spectrum (shown below the arrow). Next, an algorithm trained on input images segments structures of interest, e.g. tumor vs. stroma, demarking regions for further analysis. Finally, estimation of pixel intensity of the dye of interest (DAB) without interference from the counterstain (haematoxylin) within the demarcated region is performed.

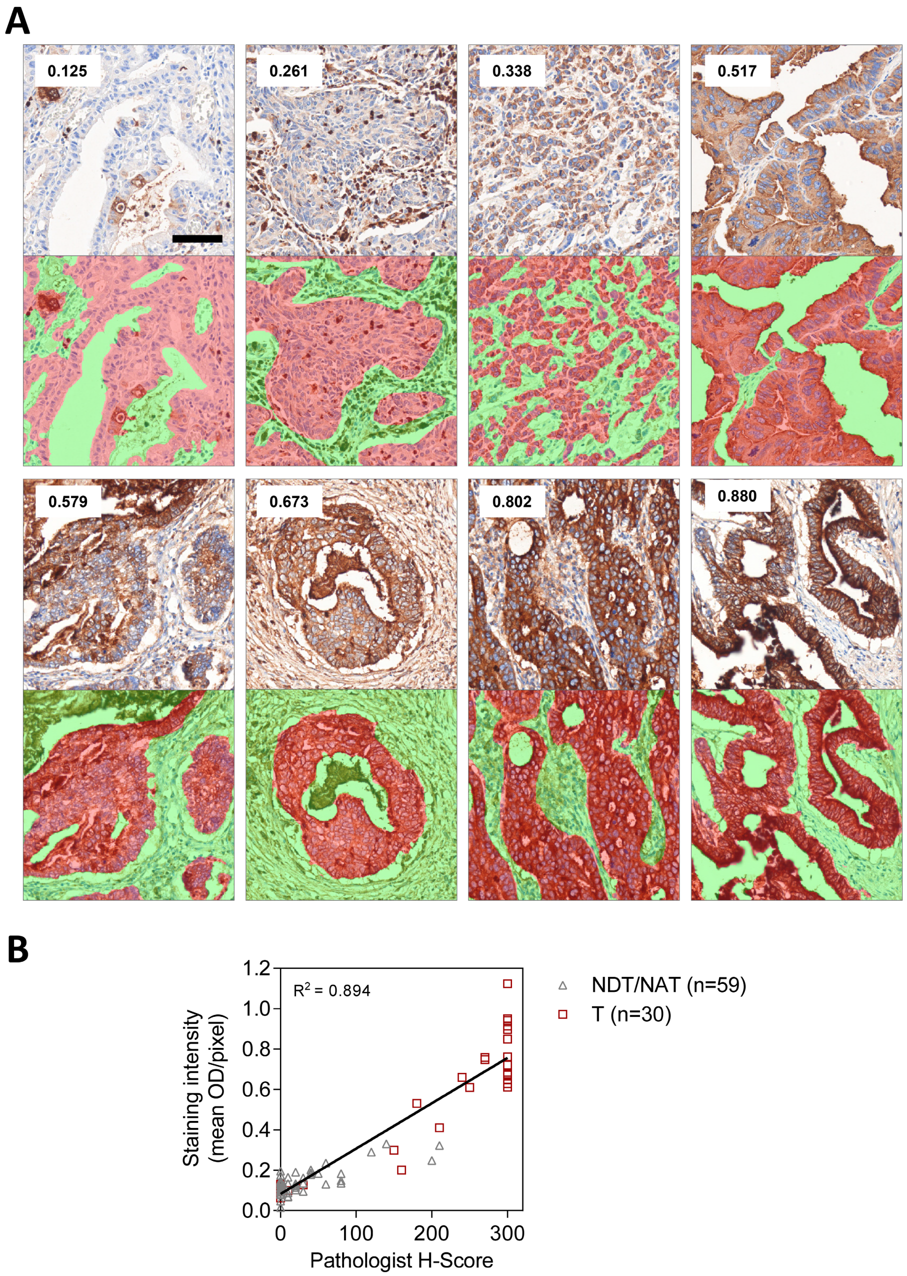

Supplementary Figure S3: A. Example IHC images of different DAB staining intensity as calculated by the Vectra 2 platform. DAB staining intensity values (mean OD/pixel) of each image are indicated in the white box and the segmented area used for readout is highlighted in red below each image. Scale bar = 50µm. **B.** Vectra 2 and pathologist scoring correlation. IHC staining against a known control marker (CEA clone II-7, Dako) was performed on a gastric adenocarcinoma tissue microarray (US Biomax, HStm-Ade090PG-01). Vectra 2 staining intensity values were plotted against pathologist H-Score of respective TMA cores. NDT = normal distant tissue, NAT = normal adjacent tissue, T = tumor.

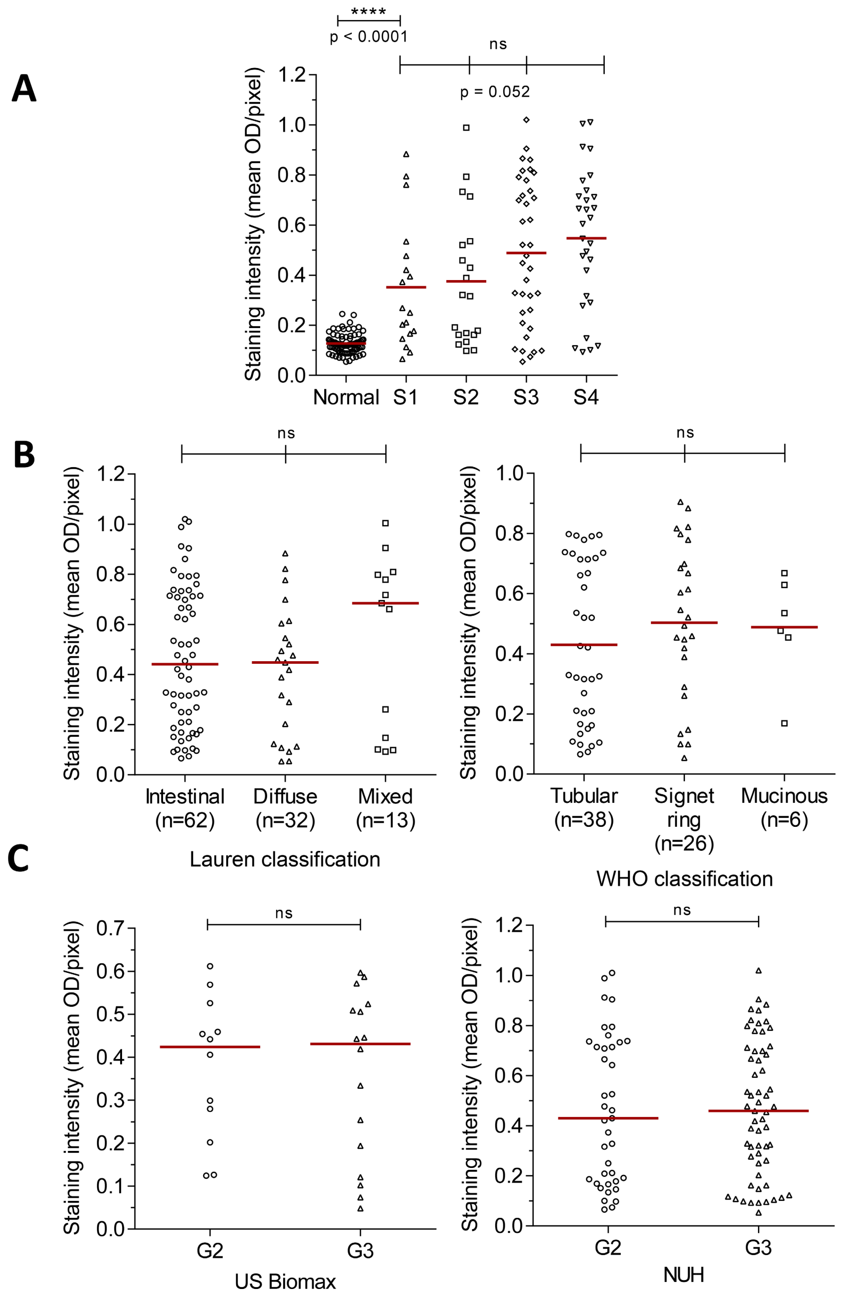

Supplementary Figure S4: A. CEACAM6 IHC stained samples from Figure 3C divided according to tumor stage. There is a significant difference in staining intensity between Normal and Stage 1 samples (unpaired t test), although overall staining intensity of tumors of different stage groups do not differ between each other. Normal n=82, Tumor Stage 1 n=18, Stage 2 n=20, Stage 3 n=37, Stage 4 n=29. Mean, one-way ANOVA. **B.** Samples from Figure 3C were divided according to tumor type in line with Lauren or WHO classification. No significant difference in CEACAM6 staining intensity is observed between tumor types. Mean, one-way ANOVA. **C.** Samples from Figure 3B (US Biomax; G2 n=12, G3 n=16) and Figure 3C (NUH; G2 n=39, G3 n=59) were divided according to tumour grade. CEACAM6 staining intensity is not associated with grade groups. G2 = moderately differentiated, G3 = poorly differentiated. G1 samples were not available for this analysis. Median, unpaired t test.

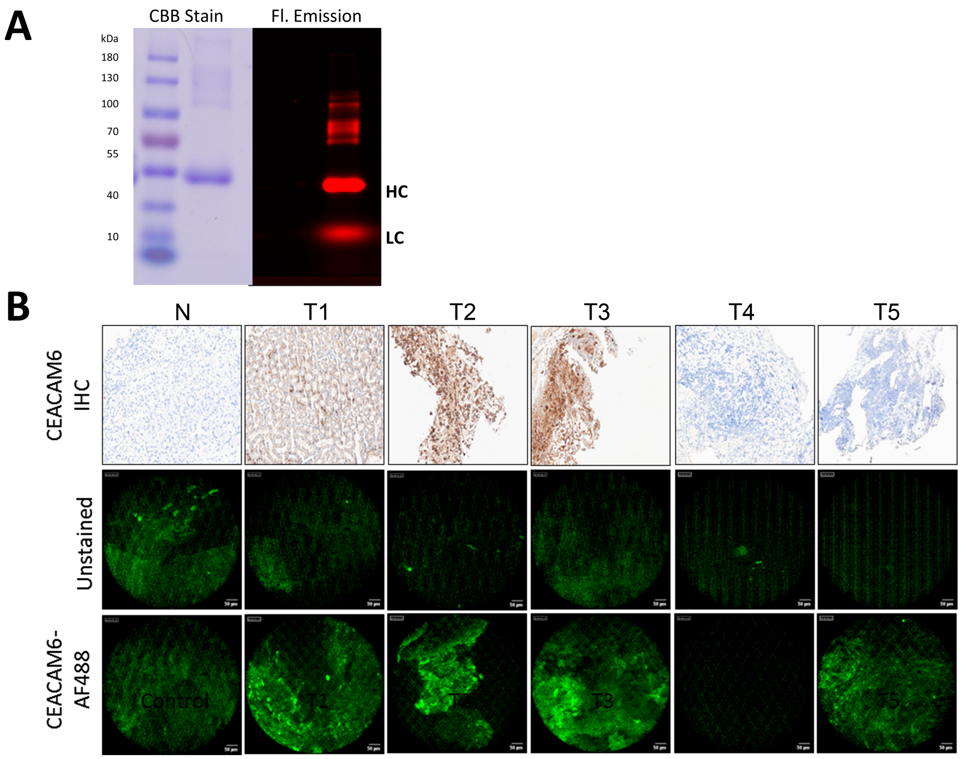

Supplementary Figure S5: A. Gel electrophoresis demonstrating Coomassie Brilliant (CBB) Blue staining and fluorescent signals emanating from a purified Alexa Fluor 488 conjugated CEACAM6 antibody. Fluorescence from AF488 is visualized using an Amersham Typhoon system. HC = heavy chain, LC = light chain. **B.** 5 xenografts (T1-5 and one mouse normal stomach tissue section, N) from the experiments described in Figure 5A-5C. The top panels shows representative images of CEACAM6 IHC staining, while the bottom panels show autofluorescece and CEACAM6-AF488 fluorescence as imaged by the Cellvizio system.

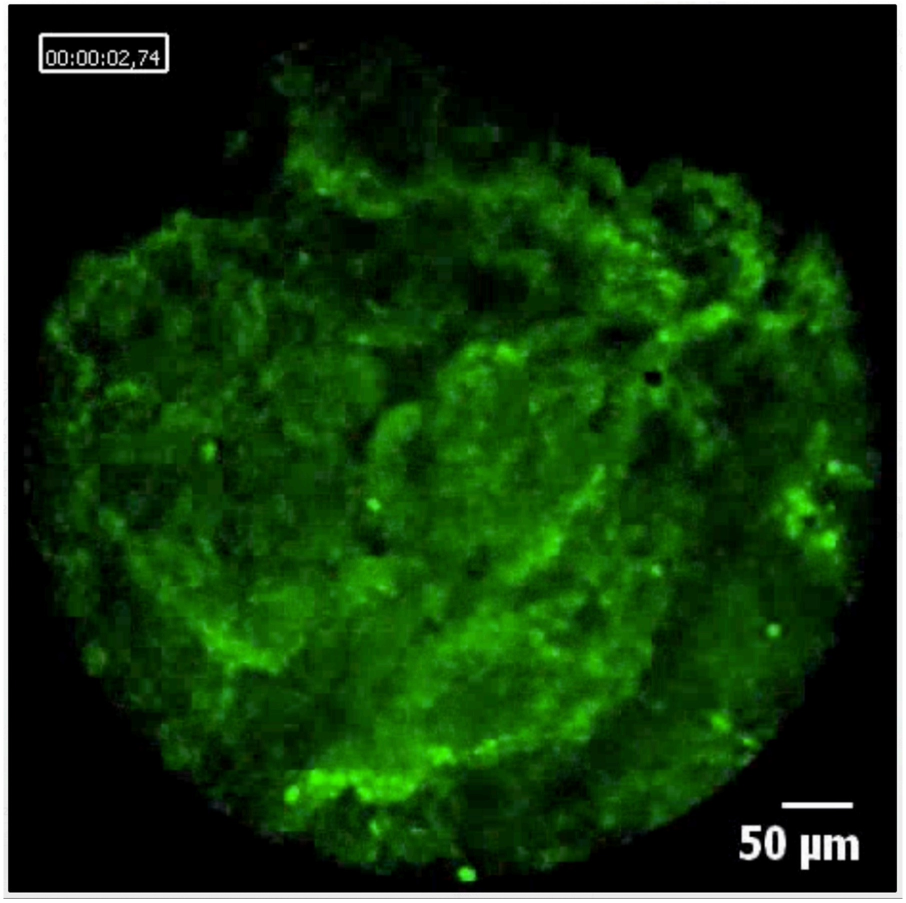

Supplementary Movie 01: Freshly resected gastric cancer xenograft tumors were stained with Alexa Fluor 488 conjugated CEACAM6 antibody, without fixation. Fluorescence imaging for AF488 was performed on these stained xenografts *ex vivo* using a Cellvizio CLE probe. This movie provides the visual effect that is observed during a simulated endoscopic assessment.
